# Concentration-Dependent Antagonism and Culture Conversion in Pulmonary Tuberculosis

**DOI:** 10.1093/cid/cix158

**Published:** 2017-02-16

**Authors:** Neesha Rockwood, Jotam G. Pasipanodya, Paolo Denti, Frederick Sirgel, Maia Lesosky, Tawanda Gumbo, Graeme Meintjes, Helen McIlleron, Robert J. Wilkinson

**Affiliations:** 1 Department of Medicine, Imperial College London, United Kingdom;; 2 Wellcome Center for Infectious Diseases Research in Africa, Institute of Infectious Disease and Molecular Medicine, University of Cape Town, South Africa;; 3 Center for Infectious Diseases Research and Experimental Therapeutics, Baylor Research Institute, Baylor University Medical Center, Dallas, Texas;; 4 Division of Clinical Pharmacology, Department of Medicine, University of Cape Town,; 5 Department of Science and Technology/National Research Foundation Centre of Excellence for Biomedical Tuberculosis Research/ South African Medical Research Foundation Centre for Tuberculosis Research, Division of Molecular Biology and Human Genetics, Faculty of Health Sciences, Stellenbosch University, Tygerberg,; 6 Division of Epidemiology and Biostatistics, School of Public Health and Family Medicine, and; 7 Department of Medicine, University of Cape Town, South Africa; and; 8 Francis Crick Institute, London, United Kingdom

**Keywords:** tuberculosis treatment outcomes, pharmacokinetic-pharmacodynamic variability, *Mycobacterium tuberculosis*, minimum inhibitory concentrations, drug–drug antagonism.

## Abstract

**Background.:**

There is scant evidence to support target drug exposures for optimal tuberculosis outcomes. We therefore assessed whether pharmacokinetic/pharmacodynamic (PK/PD) parameters could predict 2-month culture conversion.

**Methods.:**

One hundred patients with pulmonary tuberculosis (65% human immunodeficiency virus coinfected) were intensively sampled to determine rifampicin, isoniazid, and pyrazinamide plasma concentrations after 7–8 weeks of therapy, and PK parameters determined using nonlinear mixed-effects models. Detailed clinical data and sputum for culture were collected at baseline, 2 months, and 5–6 months. Minimum inhibitory concentrations (MICs) were determined on baseline isolates. Multivariate logistic regression and the assumption-free multivariate adaptive regression splines (MARS) were used to identify clinical and PK/PD predictors of 2-month culture conversion. Potential PK/PD predictors included 0- to 24-hour area under the curve (AUC_0-24_), maximum concentration (C_max_), AUC_0-24_/MIC, C_max_/MIC, and percentage of time that concentrations persisted above the MIC (%T_MIC_).

**Results.:**

Twenty-six percent of patients had C_max_ of rifampicin <8 mg/L, pyrazinamide <35 mg/L, and isoniazid <3 mg/L. No relationship was found between PK exposures and 2-month culture conversion using multivariate logistic regression after adjusting for MIC. However, MARS identified negative interactions between isoniazid C_max_ and rifampicin C_max_/MIC ratio on 2-month culture conversion. If isoniazid C_max_ was <4.6 mg/L and rifampicin C_max_/MIC <28, the isoniazid concentration had an antagonistic effect on culture conversion. For patients with isoniazid C_max_ >4.6 mg/L, higher isoniazid exposures were associated with improved rates of culture conversion.

**Conclusions.:**

PK/PD analyses using MARS identified isoniazid C_max_ and rifampicin C_max_/MIC thresholds below which there is concentration-dependent antagonism that reduces 2-month sputum culture conversion.

## Summary

The relationship between drug concentrations, minimum inhibitory concentrations, and sputum culture conversion in tuberculosis is unclear. We identified concentration-dependent antagonism between isoniazid and rifampicin that was associated with poorer 2-month sputum conversion.

The rationale for multidrug antituberculosis therapy (ATT) administered over 6 months is to ensure sterilization of both actively and slow/nonreplicating bacilli, and to prevent selection of resistant mutants. A treatment success rate of 86% has been reported in new tuberculosis (TB) cases [1]. The rate of relapse in drug-susceptible TB have been reported to be approximately 5% [2]. Interim treatment outcomes such as culture conversion by 2 months of treatment and time to culture conversion [3, 4] have been used as surrogates of outcome although, arguably, these are suboptimal measures of sterilizing activity against drug-tolerant persisting bacillary subpopulations and subsequent relapse [5, 6].

There have been hypothesis-generating in vitro studies [7–9], animal models [10, 11], and Monte Carlo simulation analyses [12, 13] predicting that variability of drug concentrations both in plasma and at the site of disease significantly affects treatment outcome [14, 15]. The relationship between bacterial growth and different antibiotic concentrations can be obtained from the pharmacodynamic parameter, the minimum inhibitory concentration (MIC). In the context of *Mycobacterium tuberculosis* (MTB), this is the lowest of a series of drug dilutions, which will limit growth of <1% (<10% for pyrazinamide) of the bacterial population under defined in vitro conditions. The pharmacokinetic/pharmacodynamic (PK/PD) parameter that best predicts microbial kill in murine and hollow fiber models for isoniazid, rifampicin, and pyrazinamide is the ratio of the 0- to 24-hour area under the PK concentration-time curve (AUC_0-24_) to MIC of the MTB strain consistent with data from clinical studies [16, 17]. Studies evaluating PK/PD predictors of 2-month culture conversion and treatment outcomes are conflicting [18–22]. This could be due, in part, to heterogeneity in geographical populations studied, the prevalence of human immunodeficiency virus type 1 (HIV-1) coinfection, dose in milligrams per kilogram, dose frequency, pharmacokinetic sampling methodology, and methods of PK/PD analysis.

Few studies have MIC data on the infecting MTB strain, necessary to calculate AUC_0-24_/MIC, maximum concentration (C_max_)/MIC, and percentage of time that concentrations persisted above the MIC (%T_MIC_). Moreover, due to the retrospective nature of many studies, not all studies had comparator pharmacokinetic data available from control patients with successful outcomes [23, 24]. Furthermore, these studies relied on concentration target ranges derived from healthy volunteers in phase 1 studies with no tuberculosis response data [25].

We assessed the role of the PK measures C_max_ and AUC_0-24_, as well as the PK/PD exposures C_max_/MIC, AUC_0-24_/MIC, and %T_MIC_ for rifampicin, isoniazid, and pyrazinamide in predicting the outcome of sputum culture conversion at 2 months in a cohort including HIV-1–uninfected and HIV-1–coinfected tuberculosis patients.

## MATERIALS AND METHODS

### Patients

Patients with GeneXpert MTB/RIF–confirmed rifampicin-susceptible pulmonary tuberculosis were recruited at Ubuntu HIV/tuberculosis clinic (site B), Khayelitsha, South Africa, as part of a prospective study (Human Research Ethics Committee approval 568/2012) assessing frequency and determinants of acquired drug resistance. The study was carried out during March 2013–July 2014, with clinical follow-up until November 2015. A subset of the patients was invited to participate in this nested pharmacokinetic study. All patients provided written consent prior to participation.

Detailed data on sociodemographic factors, past tuberculosis treatment history, and comorbidities were collected. On a single baseline sputum, bacterial load was estimated via smear grade and days to culture positivity in liquid culture media liquid (mycobacterial growth indicator tube [MGIT]). Chest radiographs were graded as extensive radiological disease in the presence of disease in both lung fields or ≥2 of 3 zones per lung, and the presence of cavitation >1 cm was also noted. Participants underwent HIV testing, CD4 lymphocyte count, and HIV-1 viral load quantification.

Antituberculosis therapy was provided as a fixed-dose combination supplied by the National Tuberculosis Control Programme (Rifafour e-275, Sanofi-Aventis; or Ritib, Aspen South Africa). Each tablet contained rifampicin at 150 mg, isoniazid at 75 mg, pyrazinamide at 400 mg, and ethambutol at 275 mg.

Weight band–based dosing was used in line with World Health Organization guidelines [26] (Supplementary Methods). Antituberculosis therapy was administered 7 days/week. At the 7- to 8-week follow-up, participants had sputum induction to ascertain culture conversion. They were classed as poorly adherent if they missed 5 or more doses of TB medication in the previous month based on either self-report and/or pill counts. They were clinically reviewed at 5–6 months and induced sputum was sent for culture to ascertain treatment completion/cure.

### Pharmacokinetics

On the day of the PK study, participants were fasting, the time of the previous dose was recorded, and all participants were observed swallowing their dose of medication. Blood draws were taken before and at 1, 2, 3, 4, 6, and 8 hours after drug ingestion after 7–8 weeks of ATT. Plasma samples were assayed for rifampicin, isoniazid, and pyrazinamide using liquid chromatography–tandem mass spectrometry methods, and plasma concentration-time data from all subjects were analyzed with nonlinear mixed-effects modeling as previously described [27] (Supplementary Methods). The free PK measures for rifampicin, isoniazid, and pyrazinamide were calculated assuming unbound fractions (f_u_) of 0.2 [28], 0.95, and 0.9 [29], respectively.

### MIC Determination

In the study cohort, the MIC for rifampicin (using concentrations of 0.03, 0.06, 0.12, 0.25, 0.5, 1 mg/L), isoniazid (0.025, 0.05, 0.1, 0.4, 1 mg/L), and pyrazinamide (12.5, 25, 50, 100, 150 mg/L) were determined in triplicate in the MGIT system with EpiCenter software (Supplementary Methods). The MICs were performed on baseline MTB isolates.

### Statistical Analyses

Sample size for this study assumed a coefficient of variation for rifampicin C_max_ (drug with greatest pharmacokinetic variability) of 40% [30]. Estimating a primary outcome rate (culture conversion at 2 months) of 70% [31], a sample size of 94 was required to detect a 25% difference in C_max_ between 2-month culture converters and nonconverters.

The median PK values for pyrazinamide were imputed for 2 patients who took only rifampicin/isoniazid on the day of PK sampling. The Kruskal-Wallis test was used to compare groups of independent variables. Unadjusted PK measures, PK measures adjusted by MIC, and clinical covariates were considered in univariate logistic regression analyses to determine predictors of 2-month culture conversion. *P* values from the univariate analyses were used to guide variable selection for the multivariate model and should be interpreted with caution in light of the potential for multiple comparisons. Clinical covariates were tested for pairwise interactions with PK parameters and the outcome of 2-month culture conversion. It was decided a priori, to include AUC_0-24_/MIC for rifampicin, isoniazid, and pyrazinamide in the multivariate model, along with the clinical covariates which were significant (*P* ≤ .2) in the univariate analysis. Stata software version 13.1 (StataCorp, College Station, Texas) was used for these analyses.

### Machine Learning

Multivariate analysis regression splines (MARS) were used to identify predictors of the probability of 2-month culture conversion. Unlike logistic regression, MARS breaks up co-linearity and complex nonlinear relationships in distinct ranges or regions of the data set, to perform an operation akin to piecewise regression with automatic examination of high-order (ie, both 1-way and 2-way) interactions [32]. The data ranges are delineated by hinges/knots, and the relationships are given as slopes in basis functions (BFs) that specify the hinges or data range reported. The receiver operating characteristic (ROC) values of the learning and test set models after 10-fold cross-validation were used to select the best model. All demographic, clinical, laboratory, and PK/PD exposure variables (C_max_, f_u_.C_max_, %T_MIC_, %T_MIC (free)_, C_max_ /MIC, f_u_.C_max_/MIC, AUC_0-24_, f_u_.AUC_0-24_, and AUC_0-24_ /MIC) were included as potential predictors in initial stepwise modeling exercises, and parameters were arbitrarily set at a maximum of 15 BFs. Thereafter, parameters in basis function format were pruned back to increase prediction on the test sample as well as to improve interpretability and parsimony (Supplementary Methods). Salford Predictive Modeler version 7.0 was used for MARS [32].

Finally, we used logistic regression to compute an adjusted odds ratio (OR) with a 95% confidence interval (CI) using thresholds identified by the MARS model.

## RESULTS

**Figure 1.**
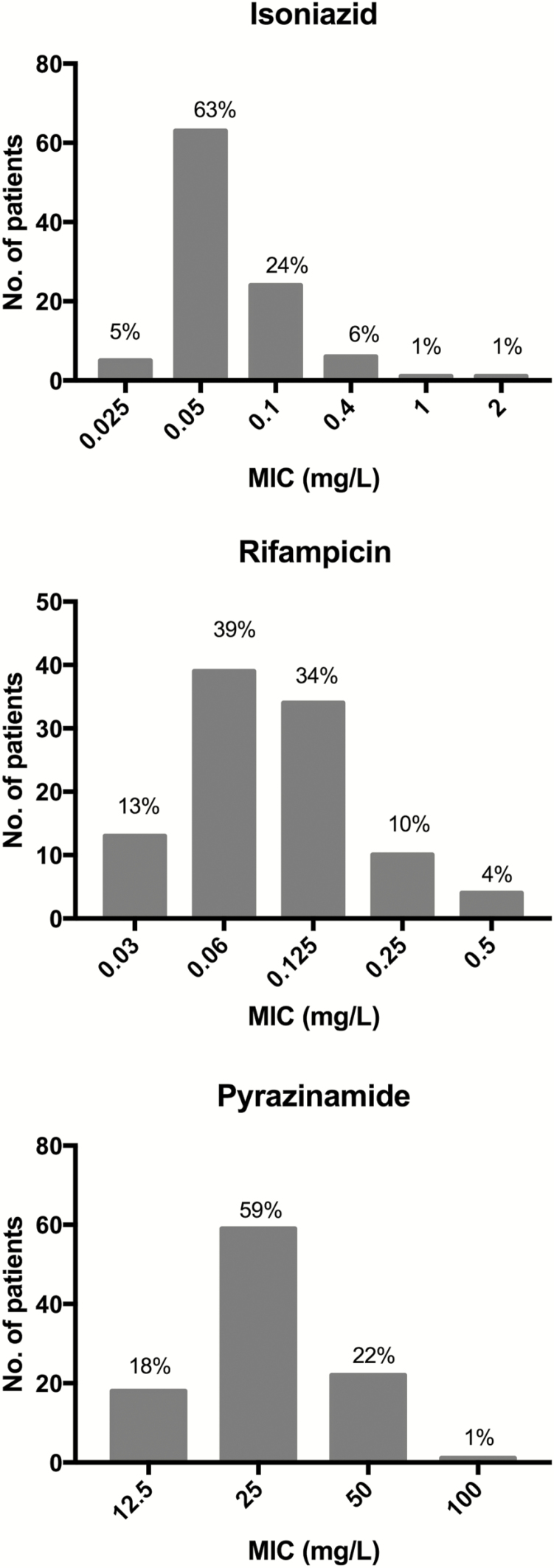
Histograms showing distributions of minimum inhibitory concentrations (MICs) in baseline *Mycobacterium tuberculosis* isolates.

Table 1 provides the clinical characteristics and treatment outcomes of the PK cohort. The median dose for rifampicin, isoniazid, and pyrazinamide was 10 (range, 7–11) mg/kg, 5 (range, 3.5–6) mg/kg, and 26 (range, 19–31) mg/kg. Of the 100 study participants, 65% were HIV-1 infected with a median CD4 lymphocyte count of 233 (interquartile range [IQR], 106–386) cells/mm^3^. The proportion on ART increased from 27 of 65 (42%) at baseline to 50 of 65 (77%) at the time of the PK study. Fifty-two percent had lung cavities present at baseline and 66% were smear positive (grading 1 to 3+), of whom 24 of 66 (36%) were graded 3+. All participants were culture positive at baseline. MIC distributions are shown in Figure 1. Culture conversion at 2 months was 77%. At the end of study follow-up, there was 1 death in the PK cohort and 3 failures and 4 relapses.

**Table 1.** Clinical Characteristics of Cohort and Outcomes

Characteristic	Whole PK Cohort (N = 100)	Culture Negative at 2 mo (n = 77)	Culture Positive at 2 mo (n = 23)
Clinical covariates^a^			
Male sex	57 (57)	43 (56)	14 (61)
Xhosa ethnicity	98 (98)	76 (99)	23 (100)
Median age, y (IQR)	33 (29–40)	32 (29–38)	40 (30–48)
Smoking history			
Current	24 (24)	17 (22)	7 (30)
Previous	27 (27)	23 (30)	4 (17)
Never	49 (49)	37 (48)	12 (52)
Alcohol consumption	37 (37)	27 (35)	10 (43)
Recreational drug use	5 (5)	5 (6)	0 (0)
Previously in prison	14 (14)	10 (13)	4 (17)
Previous mining history	5 (5)	2 (3)	3 (13)
Re-treatment	39 (39)	26 (34)	13 (57)
Type 2 diabetes mellitus	4 (4)	2 (3)	2 (15)
Median BMI at baseline (IQR), kg/m^2^	21 (19–23)	21 (19–23)	21 (20–23)
Median BMI at PK study (IQR), kg/m^2^	21.5 (20–23)	21.5 (20–23)	21 (20–23)
Median FFM at PK study (IQR), kg	45 (38–49)	44 (38–49)	47 (39–50)
HIV-1 coinfected	65 (65)	53 (69)	12 (52)
Baseline median CD4 count (IQR), cells/mm^3^	233 (106–386)	224 (101–355)	397 (216–466)
% VL <40 copies/mL at baseline	26	23	22
Median albumin at PK study (IQR), g/L	38 (34–40)	38 (34–40)	38 (34–40.5)
Median total protein at PK study (IQR), g/L	86 (79–92)	86 (78–92)	86 (83–91)
Months on ART by day of PK study (IQR)	1.32 (0–15.5)	1.3 (0.52–13.6)	14.3 (0–59.1)
Smear grading at baseline			
3+	24 (24)	13 (17)	11 (48)
2+	22 (22)	19 (25)	3 (13)
1+	20 (20)	16 (21)	4 (17)
Scanty/negative	34 (34)	29 (38)	5 (22)
Median TTD, days (IQR)	10 (7–14)	12 (7–14)	7 (6–10.5)
Extensive radiological disease at baseline	71 (71)	53 (69)	17 (74)
Cavities at baseline	52 (52)	38 (49)	14 (61)
Baseline isoniazid monoresistance	8 (8)	6 (8)	2 (9)
Median dose administered at PK study in mg/kg (range)			
Rifampicin	10 (7–11.5)	10 (9–10)	10 (9–10)
Isoniazid	5 (3.5–6)	5 (4–5)	5 (4–5)
Pyrazinamide	26 (19–31)	26 (23.5–28)	26 (23–27)
Side effects of TB treatment at 2 month review	35 (35)	24 (31)	12 (52)
Poor adherence at 2 month review as per pill counts/self-report	10 (10)	7 (9)	3 (13)
Outcomes			
5 month culture conversion (out of 83 patients who produced sputum)^b^	80/83 (96)		
Treatment failures over study duration	3 (3)		
Treatment relapse	4 (4)^c^		
Overall successful outcome (treatment cure/completion without relapse)^d^	86/99 (87)		

Data are presented as No. (%) unless otherwise indicated.

Abbreviations: ART, antiretroviral therapy; BMI, body mass index; FFM, fat-free mass; HIV-1, human immunodeficiency virus type 1; IQR, interquartile range; PK, pharmacokinetic; TB, tuberculosis; TTD, time to culture positivity at baseline; VL, viral load.

^a^At TB diagnosis (ie, baseline) unless otherwise specified as day of PK study or 2-month review.

^b^Seven defaulters, 1 transfer of care, 9 treatment completers (no sputum produced at 5 months).

^c^One relapse died and had acquired drug resistance.

^d^Defaulters assumed to have unsuccessful outcome. Transfer of care with unknown outcome not included in denominator.

**Figure 2.**
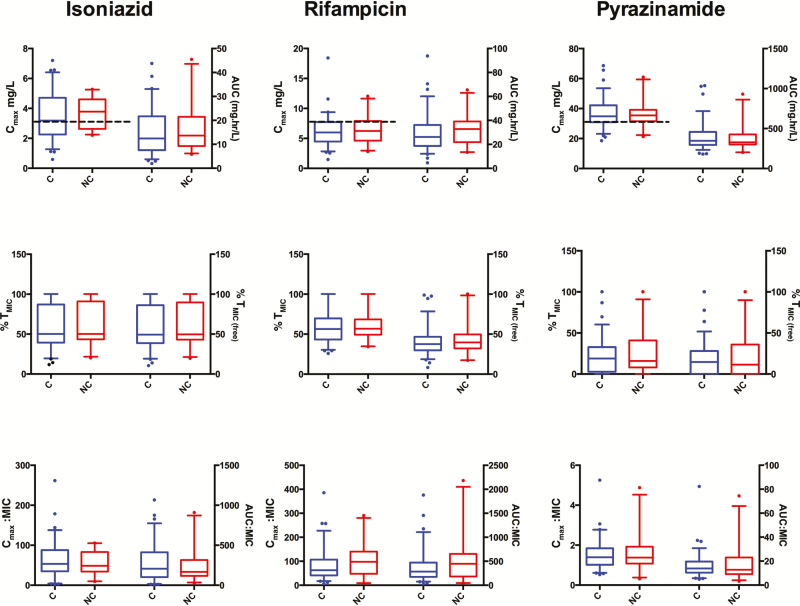
The pharmacokinetic (PK) measures maximum concentration (C_max_), 0- to 24-hour area under the curve (AUC_0-24_; with and without adjustment for minimum inhibitory concentration [MIC]) and percentage of time above the MIC (%T_MIC_), stratified by culture converter status. The box-and-whisker plots show model-derived PK measures. C_max_ and AUC_0-24_ (with and without adjustment for MIC) are plotted on the left and right y-axes. The boxes show median PK and PK/pharmacodynamic measures (and interquartile range) and the whiskers show 5th–95th percentile and illustrate considerable variability within converter (C) and nonconverter (NC) groups. The proportion of 2-month culture conversion is also shown stratified by AUC_0-24_/ MIC quartile for isoniazid (INH), rifampicin (RIF), and pyrazinamide (PZA). The dotted black line indicates current recommended thresholds for C_max_ of 3 mg/L, 8 mg/L, and 30 mg/L for INH, RIF, and PZA, respectively. There were 2 patients in whom pyrazinamide values were missing and in whom the median AUC_0-24_ and C_max_ values for pyrazinamide were imputed in the PK analysis. %T_MIC_ is the proportion of time between dosing intervals that drug concentration is above the MIC; %T_MIC(free)_ is the proportion of time between dosing intervals that unbound drug concentration is above MIC.

While there was considerable interindividual variability of C_max_, AUC_0-24,_ C_max_/MIC, AUC_0-24_/MIC, and %T_MIC_ (unbound and free), for all 3 drugs (Figure 2), on logistic regression analysis there were no statistically significant relationships between the above PK/PD parameters and the proportion culture converting at 2 months. The proportion of all patients with estimated free drug in plasma above the MIC for at least 12 hours (ie, 50% of dosing interval) was 49%, 21%, and 4% for rifampicin, isoniazid, and pyrazinamide, respectively.

For both converters and nonconverters, a significant proportion of patients had a C_max_ lower than the currently recommended guidelines for all drugs [25]. For isoniazid, 43% patients had a low C_max_ (<3 mg/L) and 6% had very low maximum concentrations (<1.5 mg/L). For rifampicin 80% had a low C_max_ (<8 mg/L) and 17% had a very low C_max_ (<4 mg/L). None of these C_max_ cutoff values for isoniazid or rifampicin predicted 2-month culture conversion and/or failure/relapse. For pyrazinamide, 53% of patients had C_max_ <35 mg/L [33] and 1% had C_max_ <20 mg/L. The cutoff of pyrazinamide <35 mg/L was not predictive of 2-month culture conversion, but did predict failure/relapse (OR, 0.16; *P* = .03). Twenty-six of 31 patients (84%) with low concentrations of all 3 drugs had culture converted at 2 months, and 4 of 31 (13%) had treatment failure/relapse compared with 3 of 69 (4%) who did not have low concentrations of all 3 drugs (*P* = .12).Table 2 shows the significant clinical predictors of culture conversion at 2 months. On multivariate analyses, 10-year increment in age (OR, 0.44 [95% CI, .24–.81]; *P* = .01), smear 3+ positivity (OR, 0.09 [95% CI, .02–.35]; *P* = .001) and drug side effects (OR, 0.17 [95% CI, .05–.63]; *P* = .01) were the only significant predictors of 2-month culture conversion.

**Table 2.** Multivariate Analysis of Clinical Risk Factors for Culture Conversion at 2 Months

Variable^a^	Univariate Analysis OR (95% CI)	*P* Value	Multivariate Analysis^b^ OR (95% CI)	*P* Value
Male sex	0.81 (.31–2.10)	.67		
10-y increment in age	0.56 (.35–.91)	**.02**	0.44 (.24–.81)	**.01**
BMI	0.97 (.89–1.06)	.48		
Re-treatment status	0.39 (.15–1.01)	.05	0.45 (.12–1.64)	.23
Smoker status				
Never	Referent			
Ex	1.86 (.54–6.48)	.33		
Current	0.79 (.26–2.35)	.67		
Alcohol use	0.70 (.27–1.81)	.46		
Ex-prisoner	0.71 (.20–2.52)	.60		
Ex-miner	0.18 (.03–1.13)	.07		
Diabetes	0.28 (.04–2.11)	.22		
Drug side effects at 2-mo review	0.39 (.15–1.01)	.05	0.17 (.05–.63)	**.01**
Poor adherence at 2-mo review as per pill counts/self-report	0.67 (.16–2.81)	.58		
INH resistance	0.89 (.17–4.73)	.89		
Smear grading^c^				
Negative/scanty	Referent			
1+	0.69 (.16–2.93)	.61	1.17 (.21–6.53, 0.85)	
2+	1.09 (.23–5.11)	.91	0.74 (.14–3.89, 0.72)	
3+	0.20 (.06–.71)	**.01**	0.09 (.02–.35)	**.001**
Time to culture positivity at baseline^c^	1.14 (1.01–1.28)	**.04**	1.16 (1.02–1.33)	**.02**
HIV status	2.02 (.78–5.23)	.15		
Log_10_ CD4	0.70 (.20–2.42)	.57		
Log_10_ VL	1.50 (1.02–2.19)	**.04**	1.51 (.86–2.67)	.15
ART at baseline	0.5 (.14–1.67)	.25		
Extensive radiological disease	0.61 (.20–1.85)	.39		
Cavitary disease	0.62 (.24–1.62)	.33		

The significance for bold values are *P* < .05. Abbreviations: ART, antiretroviral therapy; BMI, body mass index; CI, confidence interval; HIV, human immunodeficiency virus; INH, isoniazid; OR, odds ratio; TB,tuberculosis; VL, viral load.

^a^At TB diagnosis (ie, baseline) unless otherwise specified as day of pharmacokinetics study or 2-month review.

^b^Model executed inclusive of 0- to 24-hour area under the curve/minimum inhibitory concentration for rifampicin, isoniazid, and pyrazinamide as variables.

^c^Tested separately due to co-linearity.

Patients with rifampicin or pyrazinamide AUC_0-24_/MIC quartile in the second and third quartiles were more likely, on average, to achieve culture conversion at 2 months as shown in Supplementary Figure 1. Conversely, culture conversion rates were lowest in the second and third AUC_0-24_/MIC quartiles for isoniazid. Table 3 shows potential clinical predictors of conversion within the different AUC_0-24_/MIC quartiles for isoniazid, rifampicin, and pyrazinamide. A higher percentage of side effects was reported by patients with isoniazid AUC_0-24_/MIC in the highest quartile as previously reported [27]. No statistically significant interaction was found between isoniazid exposures, drug side effects, and the outcome of culture conversion. There was no association seen between pyrazinamide AUC_0-24_/MIC quartile and side effects.

**Table 3.** Distribution of Independent Variables in Patients Within Different Quartiles of 0- to 24-Hour Area Under the Curve/Minimum Inhibitory Concentration (AUC_0-24_)

Covariate	Quartile 1	Quartile 2	Quartile 3	Quartile 4	*P* Value
Isoniazid	0–116	>116–189.5	>189.5–355.8	>355.8	
Median age, y	32.6	31.1	32.3	33.7	.95
% Re-treatment	36	44	40	36	.92
% Side effects	24	32	20	64	**.004**
% Extensive radiological disease	64	88	64	68	.19
% Cavities	44	60	44	60	.46
% Smear 3+	28	24	20	24	.93
Median TTD	10	9	11	11	.94
% Poor adherence	4	16	4	16	.26
Median log_10_ VL	4.8	4.5	3.4	5.16	.22
Median log_10_ CD4	2.1	2.3	2.5	2.4	.68
Rifampicin	0–184	>184–299	>299–560	>560	
Median age, y	32.6	32.6	32.4	33.7	.68
% Re-treatment	40	44	32	40	.85
% Side effects	32	28	32	48	.46
% Extensive radiological disease	64	60	84	76	.22
% Cavities	44	48	60	56	.66
% Smear 3+	16	12	28	40	.09
Median TTD	11	13	10	9	.20
% Poor adherence	8	4	12	16	.53
Median log_10_ VL	4.6	4.7	4.5	5.2	.22
Median log_10_ CD4	2.1	2.3	2.5	2.4	.32
Pyrazinamide	0–10	>10–13.7	>13.7–19.8	>19.8	
Median age, y	31.15	32.47	34.8	33.1	.75
% Re-treatment	48	36	28	44	.48
% Side effects	28	44	48	20	.12
% Extensive radiological disease	72	88	68	56	.09
% Cavities	40	68	60	40	.11
% Smear 3+	20	24	24	28	.93
Median TTD	11	8	9	11	.87
% Poor adherence	12	8	12	8	.93
Median log_10_ VL	4.9	4.8	4.8	4.1	.88
Median log_10_ CD4	2.5	2.5	2.3	2.2	.13

The significance for bold values are *P* < .05. Abbreviations: TTD, time to culture positivity at baseline; VL, viral load.

**Figure 3.**
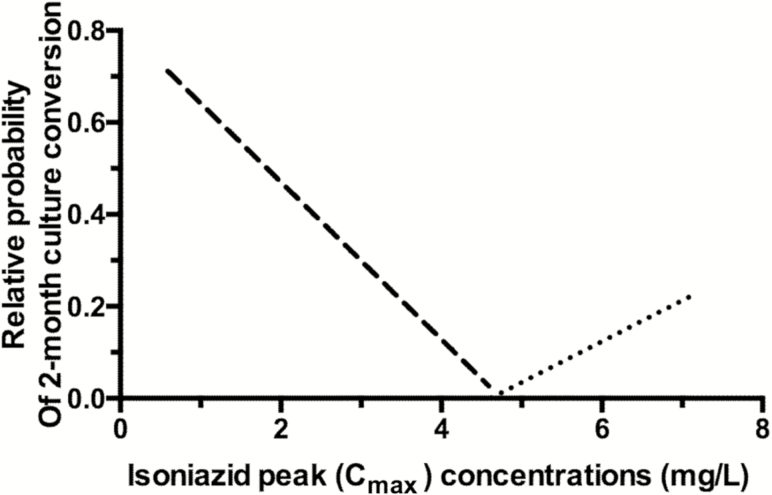
V-shaped relationship between 2-month sputum conversion and isoniazid maximum concentration (C_max_). The figure depicts the “mirror” basis function identified by multivariate adaptive regression splines with hinge at isoniazid C_max_ of 4.6 mg/L such that for patients with concentration above the threshold have an increase in probability of sputum conversion. On the other hand, for patients below the same threshold, the probability for sputum conversion increased as isoniazid C_max_ concentration decreased and has interactions with other factors (such as human immunodeficiency virus and rifampicin concentration).

Next we used MARS, to identify and rank potential predictors of 2-month conversion. The findings are shown as BFs, and the meaning of each BF is explained in Table 4. The probability of culture conversion increased with an increase in isoniazid C_max_ above 4.6 mg/L (n = 28) (as indicated by the positive coefficient of BF_1_). At an isoniazid C_max_ of 4.6 mg/L, this effect was reversed, with the probability of culture conversion increasing as isoniazid C_max_ decreased (as per positive coefficient of BF_2_). These are termed mirror BFs, the reason for which can be seen in Figure 3, where the BFs are characterized by a hinge on the value of C_max_ 4.6 mg/L and show a V-shaped relationship of isoniazid C_max_ vs the probability of culture conversion. BF_6_ shows the interaction on condition of an isoniazid C_max_ <4.6 mg/L (n = 74) and rifampicin C_max_/MIC <28 (n = 12). Below the rifampicin C_max_/MIC ratio of 28, the probability of culture conversion decreased as rifampicin C_max_/MIC decreased from 28 to 0. This effect was modified by an interaction whereby the effect of increasing rifampicin C_max_/MIC was increased as isoniazid C_max_ decreased from 4.6 to 0 mg/L. Hence, in this subset of patients, with isoniazid C_max_ ≤4.6 mg/L and rifampicin C_max_/MIC <28 (n = 9), the antagonistic effect of isoniazid on culture conversion was counteracted by increasing rifampicin C_max_/MIC. The robustness of this finding was further verified via multivariate logistic regression analysis. Among patients with isoniazid C_max_ ≤4.6 mg/L, isoniazid C_max_ was associated with reduced culture conversion (adjusted OR, 0.35 for each 1 mg/L [95% CI, .15–.80]; *P* = .01) and patients with rifampicin C_max_/MIC >28 had adjusted odds of 6.44 (95% CI, 1.02–40.54; *P* = .04) for culture conversion at 2 months (Table 5).

**Table 4.** Explanation of Basis Functions Identified in the Final Multivariate Adaptive Regression Splines Model

Basis Function	Function	Coefficient in Model	Interpretation and No. of Patients Basis Function Applies to
BF_0_	Constant/intercept	0.652	Baseline probability of sputum conversion
BF_1_	max (0, INH C_max_ – 4.6)	0.24	Where INH C_max_ >4.6 mg/L (n = 26), the probability of culture conversion was increased as an additive effect (+0.24 * BF_1_). However, at or below 4.6 mg/L, the effect of BF_1_ became zero.
BF_2_	max (0, 4.6 – INH C_max_)	0.17	The mirror image of BF_1_, basis function 2 (BF_2_), had a lower bound of 0 and was only retained when INH C_max_ <4.6 mg/L (n = 74). As the value of INH C_max_ decreased, the value of the function included increased as an additive effect (+0.17 * BF_2_). There were also some interactions with BF_3_, BF_6_ (ie, HIV-1 status and RIF C_max_/ MIC.
BF_3_	max(subset = HIV infected) * BF_2_	Nil	Basis function 3 was a dummy variable for HIV-infected patients and solely existed to interact with BF_2_ and only applied to HIV-1–infected patients with INH C_max_ <4.6 mg/L (n = 46).
BF_4_	max (0, CD4 – 190) * BF_3_	–0.001	BF4 retained its function only in HIV-infected patients with CD4 lymphocyte count >190 (n = 40). For these patients, probability of culture conversion was reduced by a small factor (–0.001 * BF_4_) as CD4+ lymphocyte count increased from 190 upwards. This function was modified by an interaction with BF_2_ where the effect size increased as INH C_max_ decreased from 4.6 to 0 mg/L.
BF_5_	max(subset = smear grade 3+)	–0.33	In those with an initial sputum smear grade of 3+, the average probability of culture conversion was decreased (–0.33 * BF_5_) (n = 24).
BF_6_	max (0, 28.00 – RIF C_max /_MIC) * BF_2_	–0.016	For patients with RIF C_max_/MIC <28 (n = 12), probability of culture conversion decreased as per negative coefficient (–0.016) as RIF C_max_/MIC decreased from 28 to 0 (–0.016 * BF_6_). This function was modified by an interaction with BF_2_, where the negative effect increased as INH C_max_ increased from 0 to 4.6 mg/L.

Abbreviations: BF, basis function; C_max_, maximum concentration; HIV, human immunodeficiency virus; INH, isoniazid; MIC, minimum inhibitory concentration; RIF, rifampicin.

**Table 5.** Multivariate Logistic Regression Analysis in Subset of Patients With Isoniazid Maximum Concentration <4.6 mg/L

Variable	Multivariate Analysis^a^ Adjusted OR (95% CI)	*P* Value
Isoniazid C_max._	0.35 (.15–.80)	**.01**
Rifampicin C_max_/MIC ≤28 C_max_/MIC>28	Referent 6.44 (1.02–40.54)	**.04**

The significance for bold values are *P* < .05. Abbreviations: CI, confidence interval; C_max_, maximum concentration; MIC, minimum inhibitory concentration; OR, odds ratio.

^a^Adjusted for all significant determinants of 2-month culture conversion in Table 2.

The optimized MARS model after stepwise elimination represented the probability of culture conversion by the equation:

$$\text{Y = 0}\text{.65 + 0}{\text{.24*BF}}_{\text{1}}\text{+ 0}{\text{.17*BF}}_{\text{2}}\text{- 0}{\text{.001*BF}}_{\text{4}}\text{- 0}{\text{.33*BF}}_{\text{5}}\text{- 0}{\text{.016*BF}}_{\text{6}}$$

Overall, the ROC for the selected model was 87% in the learn model and 66% on cross-validation, while the misclassification rates were 14% and 28%, respectively. These model performance figures are reassuring, suggesting that similar estimates could be expected in an independent sample of patients.

## DISCUSSION

The MARS model identified a potential interaction of concentration-dependent antagonism between isoniazid and rifampicin affecting outcome at the 2-month time point. This finding of concentration-dependent antagonism at the lower concentration range of rifampicin and isoniazid in the current study are consistent with murine and hollow fiber preclinical model data and antagonism in sterilizing effect rates in patients.

Chigutsa et al [19] showed in an adult South African population that an increase in isoniazid C_max_ was antagonistic when rifampicin AUC <35.4 mg × hour/L for rates of sterilizing effect based on TTD, which supported our finding of potential isoniazid antagonism in the MARS model below thresholds of rifampicin C_max_/MIC <28 and isoniazid C_max_ <4.6 mg/L. Similarly, Swaminathan et al [34] showed in Indian children with pyrazinamide C_max_ ≤38.10 and rifampin C_max_ ≤6.20 mg/L, isoniazid AUC_0–24_ >31.80 mg × hour/L led to higher proportions of children with poor outcomes. Supplementary Figure 1 shows the percentage of culture conversion stratified by AUC_0–24_ /MIC quartile for isoniazid, rifampicin, and pyrazinamide. This is consistent with findings of Almeida et al, in a mouse model of tuberculosis that showed dose-dependent antagonistic response of isoniazid on rifampicin/pyrazinamide activity, measured by colony-forming units in mouse lung [35]. The antagonistic relationship was narrowed down to the dual combination of isoniazid and pyrazinamide, which are both structural analogues of nicotinamide [35]. Grosset et al found that discontinuation of isoniazid after the first 2 days improved bactericidal activity over days 3–14 of antituberculosis treatment in mice [36]. In the hollow fiber system, coadministration of isoniazid and rifampicin at both drugs’ highest C_max_/MIC was associated with inferior microbial kill compared to administration of rifampicin after a delay of 6 hours, 12 hours, and 24 hours (coinciding with progressive fall in isoniazid concentrations), hence consistent with concentration-dependent antagonism [37].

Although this data set does not lend itself to further in depth analysis of drug–drug antagonism and synergism, the finding of drug–drug antagonism at the lower range of isoniazid and rifampicin may be a contributory factor to treatment outcomes and must be studied in further clinical studies and simulation analyses which encompass further dosing ranges of both isoniazid and rifampicin. The efficacy of isoniazid beyond its initial early bactericidal activity, should be evaluated further in the context of randomized controlled studies with appropriate follow-up and long-term treatment outcomes. Further research questions include determination of efficacy and tolerability of increased isoniazid concentrations in patients with slow *N*-acetyltransferase 2 status and the potential for staggered dosing—for example, 12-hour difference in dosing time between rifampicin/pyrazinamide and isoniazid in light of potential drug–drug antagonism. Whilst the MIC distributions were representative of populations reported elsewhere [38, 39], our cohort had good long-term outcomes and culture conversion at 2 months was 77% in liquid culture. With only 3 treatment failures and 4 relapses, the study was underpowered to study clinical and PK/PD predictors of long-term treatment outcomes. However, we did find that a pyrazinamide C_max_ <35 mg/L was predictive of unfavorable treatment outcome, consistent with findings from other groups [33, 34].

Despite significant variability of AUC/MIC for rifampicin, isoniazid, and pyrazinamide, the range of percentage of culture conversion over different AUC/MIC quartiles was limited: 64%–88% for rifampicin, 64%–84% for isoniazid, and 72%–88% for pyrazinamide. Logistic regression failed to identify a relationship between C_max_/MIC, AUC/MIC, or %T_MIC_ and the probability of culture conversion. As an example, these would average out outcomes on either side of the “V-shaped” relationship we identified, so that measures of central tendency would not differ for the range of exposures. We also note clinical covariates, which may contribute to the PK/PD trends observed. For example, there was a nonsignificant trend for patients with rifampicin AUC_0-24_/MIC >75th percentile to have a baseline sputum smear grading of 3+, suggesting that confounding by severity of the pulmonary disease may explain reduced culture conversion at this top quartile of rifampicin exposure. Although increased side effects, perhaps via reduced adherence, contributed to decreased likelihood culture conversion, this would not explain the increase in culture conversion above a certain isoniazid threshold. The latter is likely to be secondary to reversal of isoniazid-rifampicin antagonism above isoniazid C_max_ >4.6 mg/L.

This is the largest study to date reporting the effect of first-line antituberculosis drug exposures, measured by intensive sampling and inclusive of adjustment for MIC of infecting MTB strain, on the interim outcome of 2-month culture conversion in a predominantly HIV-1–coinfected cohort. There were several limitations to this study. Ethambutol exposures, which could have contributed both to the sterilizing activity of the quadruple drug regimen and also to drug–drug antagonism/synergism, were not measured. Multiple cultures were not sent during the first 2 months and, hence, time to culture conversion could not be ascertained. The binary outcome of culture conversion was via a single optimized volume induced sputum sample expectorated at week 7–8 of treatment. Lack of multiple cultures may have decreased sensitivity to determine culture conversion. However, in the context of baseline smear negative/scanty rates of 34%, the ascertained rates of conversion in MGIT cultures are unlikely to be overestimated and are comparable to populations with similar HIV-1 coinfection rates [31]. The calculated PK exposures in plasma do not necessarily equate to penetration in diseased tissue [15]. There may have been unmeasured confounders in this observational study and we may have underestimated interoccasional PK variability secondary to drug side effects and fluctuating adherence. Last, while machine-learning models are very good for generating precise hypotheses, the derived antagonistic interactions need to be tested in larger prospective studies with appropriate designs.

In summary, in this outpatient setting with a high prevalence of HIV-1/TB–coinfected patients, the majority had plasma drug exposures below accepted thresholds but nevertheless had good treatment outcomes. This was not explained by any measured clinical or programmatic factors, nor by adjusting for MIC of infecting MTB strain. We found concentration-dependent antagonism of isoniazid at the lower range of rifampicin affecting the interim outcome of 2-month culture conversion. Large studies with better biomarker models of disease response, detailed accounting for day-to-day PK variability, and further analyses evaluating the nonlinear effects of drugs in combination may further the evidence base for treatment monitoring using PK/PD measures.

## Supplementary Data

Supplementary materials are available at *Clinical Infectious Diseases* online. Consisting of data provided by the authors to benefit the reader, the posted materials are not copyedited and are the sole responsibility of the authors, so questions or comments should be addressed to the corresponding author.

## Supplementary Material

Supplementary_Figure_1

Supplementary_Methods

## Notes

***Author contributions.*** Design and conception of experiments: N. R., H. M., R. J. W., and G. M. Acquisition of clinical, MTB strain, and drug concentration data: N. R. MIC work: F. S. Compartmental PK analyses: P. D. and N. R. MARS analyses: J. P. and T. G. Analysis of data and interpretation: N. R. , H. M., J. P., T. G., M. L., P. D., R. J. W., and G. M. Writing of manuscript: N. R., H. M., J. P., T. G., R. J. W., and G. M. All authors approved the final version of the manuscript.

***Acknowledgments.*** We acknowledge the patients who participated in the study, and the clinical and administrative staff of the Western Cape Department of Health. The Division of Clinical Pharmacology at the University of Cape Town would like to acknowledge Novartis Pharma for their support of the development of pharmacometrics skills in Africa.

***Disclaimer.*** The funders had no role in the study design, data collection, data analysis, data interpretation, or writing of this report. The opinions, findings, and conclusions expressed in this manuscript reflect those of the authors alone.

***Financial support.*** This work was supported by the Wellcome Trust (grant numbers WT104803 and WT084323 to R. J. W. and WT098316 to G. M.). R. J. W. is supported by the Francis Crick Institute ,which receives its core funding from Cancer Research UK (FC00110218), the UK Medical Research Council (FC00110218), and the Wellcome Trust (FC00110218); there was additional support from the European Union (grant number EU FP7 HEALTH-F3-2012–305578) to R. J. W., the National Research Foundation of South Africa (grant numbers 96841 to R. J. W., 64787 and 85858 to G. M., and 90729 to H. M.), and the South African Medical Research Council (grant number RFA SAMRC-RFA-CC: TB/HIV/AIDS-01-2014 to G. M.).

***Potential conflicts of interest.*** All authors: No potential conflicts. All authors have submitted the ICMJE Form for Disclosure of Potential Conflicts of Interest. Conflicts that the editors consider relevant to the content of the manuscript have been disclosed.
