# Supplementary figures and images for: Concentration-Dependent Antagonism and Culture Conversion in Pulmonary Tuberculosis
Source: Clin Infect Dis. 2017 Feb 16;64(10):1350–9. doi: 10.1093/cid/cix158 (PMC5411399; doi:10.1093/cid/cix158)

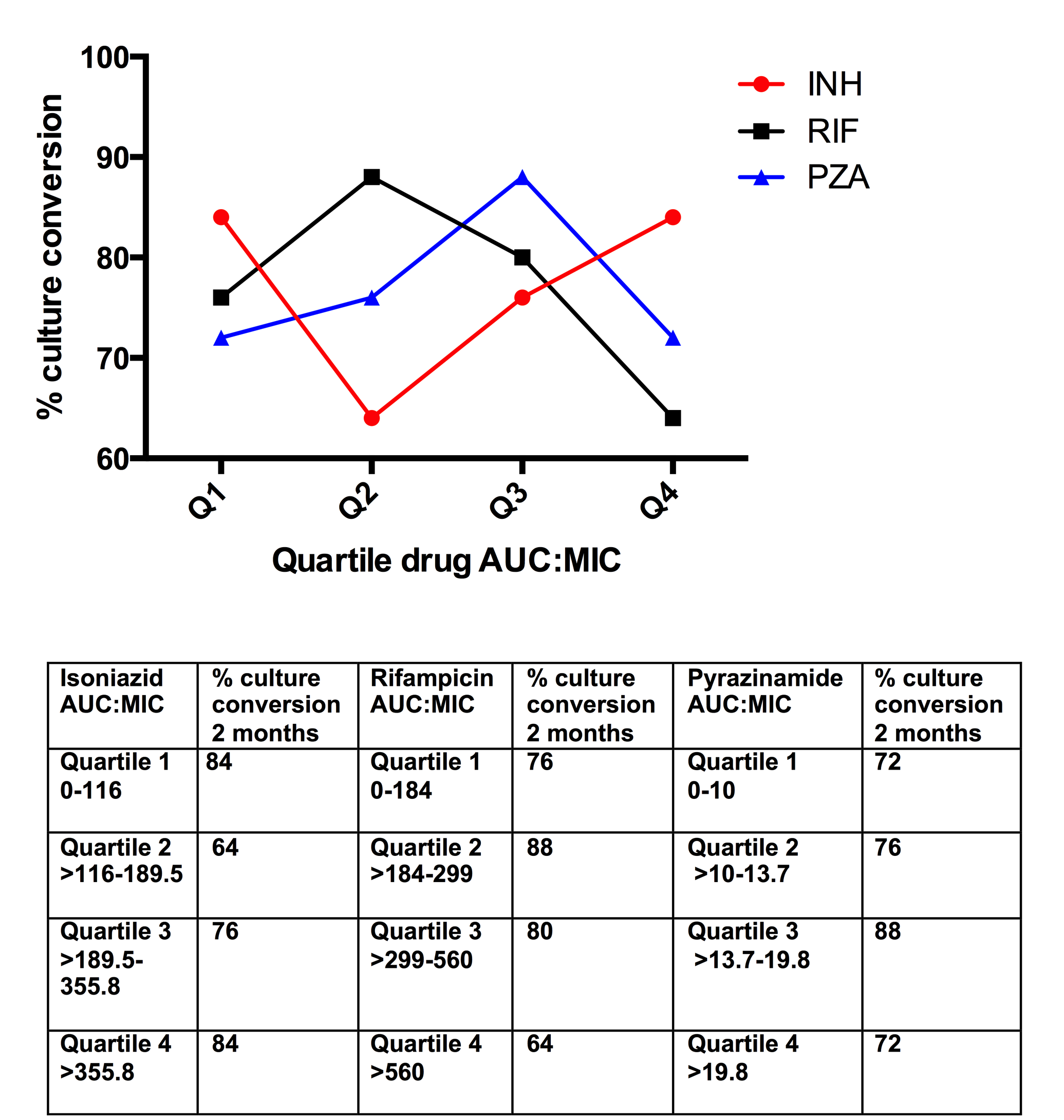

Supplement: Supplementary_Figure_1 [file cix158_suppl_Supplementary_Figure_1.png]
