# Supplementary material for: Concentration-Dependent Antagonism and Culture Conversion in Pulmonary Tuberculosis
Source: Clin Infect Dis. 2017 Feb 16;64(10):1350–9. doi: 10.1093/cid/cix158 (PMC5411399; doi:10.1093/cid/cix158)
Supplement: Supplementary_Methods [file cix158_suppl_Supplementary_Methods.docx]

**Supplementary methods**

**Antituberculosis therapy dosing and adherence assessment**

Patients weighing 38 to 55 kg (weight band 1), 55 to 70 kg (weight band 2), and above 70 kg (weight band 3) were given doses of 3, 4, and 5 tablets, respectively[1]. In keeping with the programme, patients had directly observed therapy for approximately 1 week. They were then given a month supply of medication if assessed as suitable for self-medicating by an adherence support worker. Patients were instructed to keep and bring in all their blister packs when they returned for their 7-8 week visit.

**Pharmacokinetic parameters**

The individual post-hoc PK parameter estimates from the models were used to obtain steady-state daily exposures estimates in each patient under the assumption of 100% adherence and a 24-hour dosing interval [2].

**Minimum inhibitory concentration (MIC) determination**

A MGIT 960 system with EpiCenter software was used to determine MIC according to the 1% proportion method [3]. Bacterial suspensions were made from frozen stock (-80^o^C) in MGIT 960 medium grown at 37°C. Inocula were then prepared from MGIT subcultures 2 days after the tubes flagged positive. INH and RIF were purchased from Sigma Aldrich, South Africa while the BACTEC MGIT 960 PZA Kit (Becton Dickinson Biosciences, Sparks, USA) was used for PZA. INH and PZA was dissolved in sterile distilled water (SDW) and RIF in dimethyl sulfoxide (DMSO) with subsequent dilution in SDW. Stock solutions of the drugs were prepared at concentrations 84 times higher than the highest test concentrations required. These solutions were filter sterilized and stored at -80°C in small aliquots for not longer than 6 months. Serial 2-fold dilutions were made in SDW to obtain working solutions ranging from 21 – 168 mg/L; 2.54 – 84 mg/L and 2118 – 8400 mg/L for INH, RIF and PZA, respectively. From each drug dilution, 0.1 ml quantities of INH and RIF were transferred to standard MGIT tubes containing 7.0 ml modified Middlebrook 7H9 broth base (pH 6.8) supplemented with 0.8 ml oleic acid-albumin-dextrose-catalase (OADC). The procedure for PZA was similar, apart from using MGIT 960 PZA medium (Becton Dickinson Biosciences, Sparks) at a reduced pH of 5.9. The tubes were then inoculated with 0.5 ml of the test organisms to give final 2-fold drug concentrations ranging from 0.25 – 2 mg/L, 0.03 – 1.0 mg/L and 25 – 100 mg/L for INH, RIF and PZA, respectively. A drug-free 1:100 diluted inoculum (1:10 for PZA) was included to represent the 1% critical proportion which is used to differentiate between susceptible and resistant bacilli. The interpretation of the results were based on a threshold growth unit (GU) reading of 400 by the drug-free control. Drug containing tubes with GU readings of ≥100 at the time when the drug-free control reached a value of 400 were considered resistant and those with values <100 as susceptible. The MIC was therefore defined as the lowest drug concentration that inhibits growth of more than 99% of the bacterial population. Critical concentrations of 0.1 mg/L, 1.0 mg/L and 100 mg/L were used to define resistance to INH, RIF and PZA, respectively.

**Multivariate adaptive regression splines (MARS)**

The intent of MARS is prediction, not measures of association that rely on p-vales as is common with standard statistical approaches[4]. Multivariate adaptive regression splines (MARS) is an agonistic machine-learning algorithm. MARS is a nonparametric method that breaks complex nonlinear relationships by fitting series of spline functions to estimate slopes in distinct range or regions of set of variables[5]. Basis functions, formed by mathematically combining variables or breaking others variables into two or more, are formed in a forward step by MARS. Basis functions are defined in pairs, using a knot/hinge or values of variables that defines an inflection point along the range of a predictor. Results are in a form similar to traditional linear regression while capturing essential nonlinearities and interactions.

The model is pruned back based on user set defined misclassification cost functions and penalty. MARS works by first fitting an overly large model with basis functions (forward selection) as long as they improve the fit, with no penalty for inclusion. This can lead to overfitting. The next step is backward elimination step whereby MARS identifies a basis function whose removal will have the least impact on the residual sum of squares; this pruning process is repeated with each basis function in the model.

Basis functions are thus eliminated based on generalized cross-validation error, which is the average squared residual multiplied by a penalty that is proportional to the number of basis functions in model.

In this study, the best models were selected using graphical methods, generalized cross-validation procedures and by comparing the area under the receiver operating characteristics values of the learn and test models. The optimal MARS model is the one with the lowest generalized cross validation which is a balance between goodness-of-fit and number of basis functions. In a separate study assessing pharmacokinetic-pharmacodynamic outcomes in a paediatric population [6], even when positive (computer generated variable with 100% correlation with outcome) and negative controls (random generated variables) were added, the algorithms performed very well in recognition.

References

1. World Health Organisation. Treatment of tuberculosis guidelines. Fourth edition, **2010**.

2. Rockwood N, Meintjes G, Chirehwa M, et al. HIV-1 Coinfection Does Not Reduce Exposure to Rifampin, Isoniazid, and Pyrazinamide in South African Tuberculosis Outpatients. Antimicrob Agents Chemother **2016**; 60(10): 6050-9.

3. Keller PM, Homke R, Ritter C, Valsesia G, Bloemberg GV, Bottger EC. Determination of MIC distribution and epidemiological cutoff values for bedaquiline and delamanid in Mycobacterium tuberculosis using the MGIT 960 system equipped with TB eXiST. Antimicrob Agents Chemother **2015**; 59(7): 4352-5.

4. Breiman L. Statistical Modeling: The Two Cultures. Statist Sci **2001**; 16(3): 199-231. http://projecteuclid.org/euclid.aos/1176347965.

5. Breiman L. Discussion: Multivariate Adaptive Regression Splines. Ann. Statist. 19. **1991**: 82-91. http://projecteuclid.org/euclid.ss/1009213726

6. Swaminathan S, Pasipanodya JG, Ramachandran G, et al. Drug Concentration Thresholds Predictive of Therapy Failure and Death in Children With Tuberculosis: Bread Crumb Trails in Random Forests. Clin Infect Dis **2016**; 63(suppl 3): S63-S74.
